# Evaluation of Abfraction Lesions Restored with Three Dental Materials: A Comparative Study

**DOI:** 10.3390/clinpract13050093

**Published:** 2023-08-26

**Authors:** Bogdan Constantin Costăchel, Anamaria Bechir, Alexandru Burcea, Laurența Lelia Mihai, Tudor Ionescu, Olivia Andreea Marcu, Edwin Sever Bechir

**Affiliations:** 1 Doctoral School in Dental Medicine, “Titu Maiorescu” University of Bucharest, 189 Calea Văcăreşti, 040056 Bucharest, Romania; costachel.bogdan@gmail.com; 2 Faculty of Dental Medicine, “Titu Maiorescu” University of Bucharest, 67A Gh. Petrascu Street, 031592 Bucharest, Romania; lelia_mihai2000@yahoo.com (L.L.M.); ionescu_tudor@yahoo.com (T.I.); 3 Faculty of Medicine and Pharmacy, University of Oradea, 10 P-ta 1 Decembrie, 410073 Oradea, Romania; oli_baciu@yahoo.com; 4 Faculty of Dental Medicine, “George Emil Palade” University of Medicine, Pharmacy, Science and Technology of Targu Mures, 38 Gh. Marinescu Street, 540142 Targu Mures, Romania; bechir.edwin@gmail.com

**Keywords:** abfraction, direct restorations, mouthguards, modified USPHS criteria, evaluation

## Abstract

Background: Abfraction lesions are manifested as damage to hard tissues in the cervical area of dental crowns. The study aimed to assess the direct restoration of abfraction lesions according to the modified United States Public Health Service (USPHS) criteria for 24 months. The restorations were accomplished with Fuji Bulk–GC, Omnichroma Flow-Tokuyama, and Beautifil^®^ II–Shofu dental materials, and the therapy was or was not associated with wearing thermoformed mouthguards. Methods: From the 53 selected and analyzed patients (*n* = 53), 28 patients (with restorations of abfraction lesions) belonged to the 1st group and 25 patients (with 105 restorations, who also wore mouthguards) belonged to the 2nd group. Blind determination assessments were effectuated at baseline and after 2, 6, 12, 18, and 24 months. Results showed that, regardless of the rating score, there are no significant statistical differences in the evaluation criteria between the two groups of patients Conclusions: For each material, the scores of USPHS criterion presented good clinical performances after 24 months, with no significant statistical differences between the fillings and the applied therapy in the two groups of patients.

## 1. Introduction

Non-carious cervical lesions (NCCLs) are represented by irreversible damage to the dental hard tissues (enamel, dentin, and cement) at the level of the cement–enamel junction (CEJ) [1,2]. The CEJ is stable over time [3] and presents four types of relationships: the dental cement overlaps the enamel; “edge-to-edge” junction; exposed dentin due to the existence of a gap between the enamel and cement; enamel covers the cement [4]. In areas affected by NCCLs, the tertiary reparative dentin presents sclerotic dentinal tubules, occluded by mineral precipitations of hydroxyapatite in other crystallographic forms. NCCLs are categorized as abrasion, attrition, erosion, abfraction, and biocorrosion [5,6]. NCCLs have a considerable prevalence today, and are actually related to people’s lifestyles and age [7]. 

Abfraction lesions are understood as having a multifactorial etiology, and represent an irreversible pathologic damage to the tooth’s hard tissue [2,8]. They are localized along dental crowns’ labial or buccal cervical zone, especially in premolars and the cervical area of incisors, canines, and molars [9,10,11]. According to El-Marakby et al. [12], abfraction lesions are frequent in the population over 40 years of age. Many researchers suggested the connection between the existence of non-physiological compressive occlusal forces, parafunction (e.g., bruxism), abrasion, and erosion [2,8,13,14,15,16,17]. Clinical appearances of abfractions are manifested as V- or wedge-shaped (with obviously delimited internal and external angles), or as C-profiled, forming lesions with circular walls. After the progression and the associated etiological factors, abfractions can be more profound than extensive. Sometimes they can be situated under the gingival margins [12]. The destruction of all hard dental tissues is progressive [18]. By depth, abfraction lesion are divided in three types: (a) lesions which penetrate only the enamel; (b) enamel and dentin lesions; and (c) lesions that penetrated the pulp tissue [19]. 

To diminish the initiation and progress of abfractions through the elusion/control of improper occlusal forces, the use of night guard appliances has been suggested [2]. Several kinds of oral parafunctional habits can affect the oral system, including bruxism [20,21,22]. Augmented action of the masticatory muscles (which can develop high biting forces of 400–1100 N) can determine the appearance of occlusal overloads and abfractions [23]. The therapy for occlusal overloads should include the use of intraoral devices (occlusal splints, thermoformed mouthguards) to protect the teeth and restorations from possibly excessive forces [24,25]. 

The aim of the study was to assess the direct restorations of abfraction lesions according to the modified United States Public Health Service (USPHS) criteria for a period of 24 months. The restorations were carried out with three types of dental materials, and the dental therapy was or was not associated with wearing thermoformed mouthguards.

## 2. Materials and Methods

The research was accomplished by implementing the ethical principles of the Declaration of Helsinki, good clinical practice, and GDPR practices regarding data protection. The research protocol was authorized by the Ethics Committee of the Dental Medicine Faculty, Titu Maiorescu University of Bucharest (No. 7 of 14.01.2019). All selected patients were notified regarding the study’s demands, and only those who willingly accepted the demands were admitted. The stages of the study and the necessity of monitoring were explained to each selected subject. Written informed consent was acquired ahead of the beginning of the study. The study was carried out in the Clinics of the Dental Medicine Faculties, from November 2019 to November 2022, with an 18-month intermission due to the context of the COVID-19 pandemic.

Before the selection of patients for the study, all the authors followed three practical calibration sessions regarding the precision of patients’ anamneses, clinical examination, and diagnosis; correct determination of abfraction’s degree; suitable use of the devices in conformity with the protocol (EMS Piezon, EMS Air-Flow, etc.); educational standards for adequate oral hygiene; proper application of all filling materials; the use of the same material and technique in the achievement of the thermoformed mouthguards; and the reliability of the study and acquired results. The patients selection for the study was performed by three authors, the treatment itself was performed by two other authors, and the follow-up observations were performed by the authors who were not involved in the other two stages of the study. Those who performed the direct restorations of the abfraction lesions were blinded to those who subsequently monitored the patients. The patients did not receive information regarding the location of each type of dental material applied. 

A comparative study of the fillings (aspect, maintaining, and esthetical characteristics), applied as monotherapy for the 1st group of patients or applied in association with thermoformed mouthguards in 2nd group of patients, was realized. All patients included in the study were selected in conformity with the same inclusion and exclusion criteria. The trial has a blinded study design. Detailed anamnesis (with personal data, employment, the existence/absence of any allergies, nutritional habits, parafunctions/vicious habits, acute and/or chronic illnesses, etc.), accurate clinical examination (inspection of the oral cavity, assessment of oral hygiene status, localization and degree of abfraction lesions, differential diagnosis to other NCCLs types, etc.), and X-ray examinations (orthopantomograms or/and intraoral radiographs) were obtained to assess the eligibility of patients. The inclusion criteria in the study are presented in Table 1 and the exclusion criteria in Table 2.

Initially, 84 subjects were assessed for eligibility, but only 71 patients were enlisted in the study. The included patients were initially divided in 2 groups of 36 and 35 patients, but after being informed of the conditions of the study, the number of patients that agreed to wear the thermoformed mouthguards (second group) was less than that of the first group of patients. Patients who did not want to wear thermoformed mouthguards, but accepted the other requirements of the study, were transferred to the first group of patients. So, the 1st group of patients (*n* = 38) received esthetic fillings for the abfraction lesions, and the 2nd group (*n* = 33) were treated with esthetic fillings and thermoformed mouthguards. Of the 1st group of patients (*n* = 38), 1 patient was withdrawn voluntarily during the study, and 2 patients were excluded for lack of compliance. Of the 2nd group of patients (*n* = 33), 1 patient was withdrawn voluntarily. During follow-up, 14 patients were lost, out of which 3 patients belonging to the 1st group and 4 belonging to the 2nd group were withdrawn voluntarily and the rest were excluded for lack of compliance. 

Finally, the number of evaluated patients was as follows: (*n* = 53), 28 for the 1st group (esthetic fillings of the abfraction lesions), and 25 for the 2nd group (esthetic fillings of the abfraction lesions and the use of thermoformed mouthguards). The age interval of the remaining and evaluated patients (53 patients, 29 male and 24 female), was 38–59 years (means 48.5 ± 10.5 years). The total number of studied abfraction lesions was 219, of which 114 (61 maxillary and 53 mandibular) belonged to the 1st group and 105 (57 maxillary and 48 mandibular) belonged to the 2nd group of patients (Table 3).

### 2.1. Educational Measures for Adequate Oral Hygiene Technique 

Oral hygiene training was performed with all selected patients 2 weeks before the restorative procedures. The presence/absence of dental plaque and calculus was revealed without any score with GC Tri Plaque ID Gel. Proper use of tooth-cleaning tools was presented and then practiced (modified Bass technique for tooth brushing [26] twice a day for three minutes, with Sensodyne Pronamel toothpaste and toothbrush, Pronamel Daily mouthwash–GSK). The scaling was performed with the EMS Piezon and EMS Air-Flow Master Units.

### 2.2. Dental Materials

The dental materials used for the restoration of abrasions were represented by GC Fuji Bulk (GC Corporation, Tokyo, Japan), Omnichroma Flow (Tokuyama Dental Corporation, Germany), and Beautifil^®^ II (Shofu Dental, Kyoto, Japan). Fuji Bulk capsules (FuB) are a high-viscosity, rapid-setting, and self-curing universal shade glass ionomer cement, containing ultra-fine glass particles embedded in a higher-molecular-weight matrix of polyacrylic acid [27,28]. Omnichroma Flow (OmF) is a syringeable low-viscosity light-curing composite restorative material with a chameleonic effect. The composite matrix contains uniformly sized (260 nm) globulous filler radiopaque particles [29]. Beautifil^®^ II (BeaII) is a bioactive, nanohybrid, fluoride-releasing, light-cured giomer. It is based on the technology of pre-reacted glass filler particles, which are embedded in the resin matrix [30]. 

### 2.3. Filling Technique

The color shade of each tooth with abfraction lesions was recorded before their restoration. A disposable saliva ejector was attached to the suction pump and cotton rolls were used to maintain a dry operating area. The restorations of the abfractions was performed without the use of gingival retraction cord, infiltration anesthesia, or rubber dam.

Every patient benefited from at least four restorations, carried out with Fuji Bulk capsules, Omnichroma Flow, and Beautifil^®^ II. The materials were applied according to manufacturer’s instructions. 

The selective-etch bonding technique for mineralized enamel was used. The abfraction area was dried without desiccation. GC cavity conditioner for the restored abfraction lesions was used. Then, one single layer of adhesive was applied depending on the filler. For GC Fuji Bulk, Universal Bond adhesive was used; Tokuyama was used for Omnichroma Flow; and FL-Bond II adhesive was applied for the Beautifil^®^ II giomer. The restorative materials were placed in bulk and sculpted. Cervical matrices (Cure-Thru Clear Cervical Matrices, Premier Dental) were applied over the abfraction area and firmly pressed onto the surrounding enamel surface. A Woodpecker O-Light Curing Light was used for light curing. The restored area was defined with a flame-peak diamond bur, then finished with a rough disc (Sof-Lex Contouring and Polishing Discs-3M) and polished with medium and fine polishing cups (FlexiCups, Cosmedent, Chicago, IL, USA), silicon carbide brushes and polishing paste (Enamelize, Cosmedent, Chicago, USA). The patients were advised not apply any pressure on the restored teeth for 2 h.

### 2.4. Thermoformed Mouthguards

Thermoformed mouthguards were used to decrease the non-axial loading on the teeth. Thermoforming devices were vacuum manufactured out of a polymeric sheet plasticized by heating on a mold. After the depletion of the air, the specific shape was obtained, followed by processing and polishing [31]. Erkoflex soft-elastic thermoplastic ethylvinylacetate polymer sheets with 1.5 mm thickness (Erkodent, Erich Kopp GmbH, Pfalzgrafenweiler, Germany) were used.

### 2.5. Assessments Criteria of the Restorations in Treated Abfraction Lesions

The evaluation criteria used in this study for scoring the restorations accomplished in the abfraction treatment were represented by the modified United States Public Health Service (USPHS) criteria for direct clinical evaluation of the restoration [32,33]. Examinations were performed by visual inspection, the use of an explorer, or both (Table 4).

Both groups of patients benefited from the same dental treatment protocol, except for the thermoformed mouthguards, which were applied only in the 2nd group. The materials used for the direct restorations were randomly located, in order to achieve the blinding requirement of the study (only those who made the fillings knew which dental material was used and for which abfraction lesion). Two dentists restored the abfraction lesions. 

Monitored patients (*n* = 53):

- 1st group (*n* = 28) with 114 aesthetic fillings for abfraction lesions (61 maxillary and 53 mandibular), where 38 fillings with GC Fuji Bulk were performed, 37 with Omnichroma Flow, and 39 with Beautifil^®^ II;
- 2nd group of patients (*n* = 25), with 105 esthetic fillings for the abfraction lesions (57 maxillary and 48 mandibular), where 34 fillings were accomplished with GC Fuji Bulk, 36 with Omnichroma Flow, and 35 with Beautifil^®^ II materials; also, the treatment for the patients of this group was associated with the use of thermoformed mouthguards. Of the 118 maxillary abfractions, the most restored teeth were premolars. Maxillary abfractions were represented by 84 premolars (71.18%) and 34 canines (28.81%). Mandibular abfractions were all observed in premolars (101). Of the total 219 abfractions, 185 (=84.47%) were located at the premolars.

**Figure 1:**
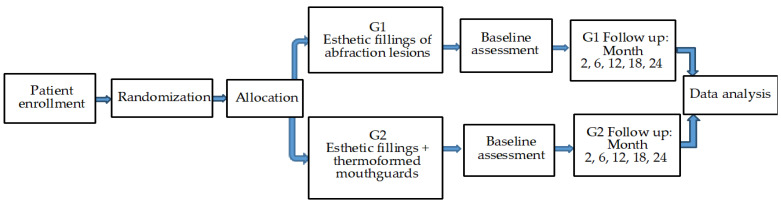
Visit schedule of assessments.

For all the assessments of the researched criteria, the same type of dental mirror, probe, air cannula, dental unit, and dental loupe (Galilean-Style Dental Surgical Medical Binocular loupe, 3.5X magnification—Gain Express) were used. All parameters were recorded during evaluations by using a standardized case report form. Inconsistencies in the scores of the two evaluating dentists were solved by consensus during the assessment sessions. Patients were blinded regarding the used dental materials and their location. The assessments were accomplished by two dentists (other than the ones that treated the abfraction lesions) at baseline (after performing the fillings) and in follow-up assessments (2, 6, 12, 18, and 24 months later) (Figure 1).

The comparative analyses of the fillings and the behavior of the dental materials used in both groups of patients were performed in assessnents and after 24 months of follow-up. 

### 2.6. Statistical Analyses

All statistical analyses were conducted in the IBM software SPSS 24 (Armonk, NY, USA). 

Tests for differences among the two types of therapies applied in the first and second group of patients for each qualitative criterion used in the study (A, B, C, cumulative), each direct restorative material used (FuB, OmF, Beall), and every aspect (color match/stability, marginal discoloration, surface texture, anatomical contour/form, marginal adaptation/integrity, secondary/recurrent caries, retention of restoration/fracture) were carried out through means of the Pearson’s Chi Square test. In all cases, the significance level was considered 0.05; otherwise, it was specified. Missing corresponding *p*-values are due to lack of data.

## 3. Results

**Figure 2:**
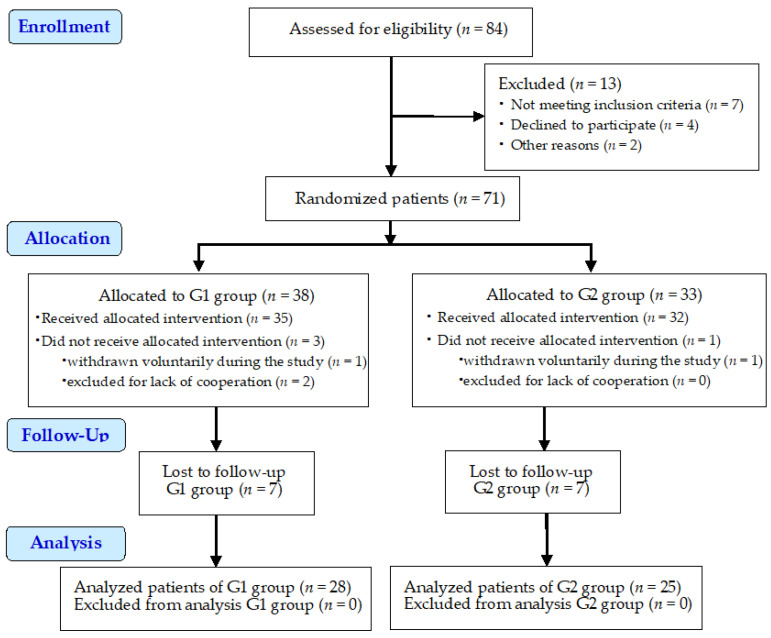
Study flow diagram.

The flow diagram of the study is presented in Figure 2.

### 3.1. Clinical Results

The restorations of abfraction lesions in this study were accomplished with three types of direct restorative materials (a glass ionomer cement, Fuji Bulk; a composite resin with low viscosity, Omnichroma Flow; a nano-hybrid aesthetic giomer, Beautifil^®^ II). The evaluation scores obtained by using the modified USPHS criteria of restorations referring to the 1st group of patients are presented in Table 5, and that for the 2nd group in Table 6. 

The comparative results of the characteristics of the investigated restorations according to the modified USPHS criteria highlighted the following:

- The data noted in Table 5 and Table 6 shows that, in concordance with the studied criteria, in descending numerical order of the B and C scores, the most numerous changes appeared in the abfractions restored with BeaII, followed by FuB and OmF;
- Also according to Table 5 and Table 6, the data shows that numerically the B and C scores of the criteria followed in this study were lower in the second group of patients, who were treated with the associated therapy (fillings and thermoformed mouthguards);
- We mention that teeth with abfraction that were restored with one of the three types of materials did not require endodontic treatment.

Differences between the two groups of patients for each score level were checked, including the cumulative one since the interpretation can be divided for each variable and/or criterion. 

The occurrence of new abfraction lesions was not observed in either group of patients during the assessments.

### 3.2. Statistical Results

Table 7 presents the comparative results regarding the studied criteria for the dental materials used for the rehabilitation of abfractions, with acceptable (B), unacceptable (C), and cumulative undesirable scores (B + C), as well as statistical results for both patient groups at all assessments.

The statistical results showed that, regardless of the rating score, there were no significant differences in the evaluated criteria between the two groups of patients, regardless of the restorative material used or score level. All of the patient’s teeth were rehabilitated with the same three types of restoration materials, by using the same technique, and the examination of their behavior during the two years of the study was performed by using the double-blind method.

## 4. Discussion

Fuji Bulk self-cure glass ionomer cement offers a balance between its restoring role and its protection of tooth hard tissue from acid challenges. It is indicated for use where acid resistance, less solubility, bulk curing, and speed of insertion represent priorities above aesthetics [27,28]. Light activation of conventional glass ionomer cements (GICs) permits the formation of supplemental cross-links [34]. The disadvantages include great polymerization contraction and cytotoxicity [35,36]. After Faridi et al. [37], the strength of the GICs was raised for the encapsulated form when compared to the manually mixed form. Through their bioactivity and fluoride-releasing properties, GICs prevent the occurrence of recurrent caries [38,39]. GICs with pre-reacted fillers of composite resins (CRs) exhibit reduced remineralization potential compared to traditional GICs, but are capable of inhibiting the demineralization of the enamel [40]. The incorporation in GICs of bioactive glasses can induce the precipitation of fluorapatite crystals in the demineralized dentin layer [41,42] and remineralization [41,43]. The use of giomers obtained through S-PRG technology determines the remineralization of the tooth’s hard structures, and they have a role in antibacterial activity [44,45,46,47,48]. 

Currently, CRs are the principal restorative materials due to their proper properties (aesthetics, reasonable durability) and relatively low cost, but their prime cause of failure is represented by secondary caries [49,50]. Omnichroma Flow is a one-shade flowable dental composite, with uniformly dimensioned supra-nano globulous filler, that uses Smart Chromatic Technology. They have high finish potential, increased stain resistance, low polymerization shrinkage, and high flexural and compressive strength. This composite is indicated for use in direct restorations in small cavities, cavity bases, cavity liners, and restoration of porcelain/composite [51,52].

Beautifil^®^ II is a highly filled bioactive nano-hybrid dental CR containing SPRG-filler, and is resistant to acid attacks [53]. Bezerra et al. [54] evaluated the clinical behavior of GICs and CRs used in NCCL. CRs exhibited better outcomes than the conventional GICs in marginal adaptation and marginal discoloration, but GICs had better clinical results than CRs in the retention of the restorations. Yeo et al. [55] concluded that bulk-fill composite resins present significantly higher flexural strength than conventional composite resins. Boing et al. [56] compared the color match and the retention loss of GICs to RCs of restored NCCLs in a 2-year follow-up, and observed that in GICs the retention loss was lower compared to that of RCs, increased rugosity was noticed in GIC restorations, and the color in RCs was superior compared to GICs. In Heintze et al. [57], a meta-analysis of clinical outcome parameters in RC and GIC posterior restorations, the applied nanohybrid resins were not greatly superior to the hybrid or microhybrid RCs, but the restorations made with compomers and GICs demonstrated significantly shorter longevity. The survival rate after 10 years for RC restorations decreased to 85–90% (with no significant difference between hybrids, microhybrids, and nano-hybrids).

Ferracane [58] considers that many factors are responsible for the occurrence of marginal leakage and recurrent caries, and the clinical outcomes are different from in vitro results. Serin-Kalay [59] evaluated five commercially available bulk-fill RCs after their immersion in water and coffee, and bulk-fill dental materials presented a higher susceptibility to discoloration than conventional RCs. The esthetic features of GICs represent a subject that impedes their use in the case of patients with increased esthetic requirements [60]. 

At the beginning of this study, oral hygiene training was performed in the dental office for all the patients before completing the restorative procedures, until all of them applied the correct technique. The presence/absence of dental plaque and calculus was highlighted with GC Tri Plaque ID Gel without scoring. Patients brushed their teeth with the modified Bass technique twice a day for three minutes with 1 cm of Sensodyne Pronamel fluoride toothpaste with the Sensodyne Pronamel medium-hard toothbrush and micro-fine bristle heads, and rinsed their mouths daily for 1 min with approximately 10 mL of Pronamel daily mouthwash (GSK House, Brentford, UK). These dental hygiene procedures were verified during the study, and thus the health of the oral tissues was maintained. All selected and included patients had to show a Silness and Löe index score of 0 in the plaque index (no plaque is in the area adjacent to the gingiva) and in the gingival index (absence of inflammation, healthy gums). Teixeira et al. [61] studied the gingival tissues near NCCLs after their restoration with dental composites finished with two type of polishing techniques. Their results did not present differences in the examined periodontal parameters after 6 months. Nassar et al. [62] evaluated the biocompatibility of the restored abfractions with CRs (using different fillers finished with diverse polishers) for 3 months in patients with teeth affected by periodontal conditions and with diabetes mellitus. They found that the CRs with nanoparticles presented clinical biocompatibility, regardless of the type of polisher used.

The periodontium phenotypes, represented by the gingival and by the underlying osseous tissues, present a large variability and significantly different responses to injuries, and require specific treatment [63,64,65]. The treatment of cervical lesions can induce the occurrence of irritating lesions and chronic illness of the periodontium, especially when they are located at the level of the free gingiva, due to the technique applied in the isolation of the prosthetic field in filling the lesions and in the inappropriateness of the marginal sealing technique [66,67]. In this study, the abfraction restorations were carried out without the use of a gingival retraction cord or rubber dam on account of the fact that the cervical margins of the abfraction lesions were located at the level of the free gingival margin, so the structure of the marginal periodontium was not damaged.

Clinicians should choose suitable dental materials based on the proper features/properties and indications of an individual’s clinical state. Before any restorative intervention on abfraction lesions, the correspondence between the patient’s age and if the lesions endanger the tooth vitality and their function should be concluded because, in a significant number of cases, patients do not complain about their abfraction lesions as they are often painless and not remarkably visible. So, it is necessary to monitor these lesions at regular intervals, without any dental intervention, where no critical manifestations are observed, and where the lesions are shallow (less than 1 mm). The abfraction lesions should be monitored every 6–12 months and should accompany regular oral hygiene surveys [2]. 

Research about the biocompatibility and the specific properties of restorative materials, including the proper GIC and RC characteristics, filler particle features, surface changes, antimicrobial properties, remineralization, and wear behavior in the patient’s oral cavity, is called for and should be developed in the future [49,68].

The clinical relevance of the research, within its limits, consists of the results obtained for the investigated criteria, which suggests valuable outcomes for each of the restorative dental materials used (Fuji Bulk capsules, Omnichroma Flow, and Beautifil^®^ II) in the restoration of abfraction lesions.

## 5. Conclusions

Within the study’s limitations, it has been shown that the scores for each USPHS criterion presented relevant clinical behavior after 24 months, with no significant statistical differences between the used restorative materials and the applied therapy in the two groups of patients.

## Figures and Tables

**Table 1:** Inclusion criteria for patients in the study.

Patients between 38–59 years of age
Accomplishment of correct oral hygiene
At least four abfraction lesions on the same dental arch (maxillary or mandibular), at the level of upper or lower premolars or upper canines, for greater blinding regarding the location of the dental material used
Abfraction lesions in the second stage
Teeth without dental and/or periodontal conditions
Absence of manifest occlusal wear signs, parafunctional habits, and patients without dento-maxillary anomalies
Non-existence of prosthetic rehabilitated or absent antagonistic teeth
Patients in a good health state that allows for dental therapy
Non-/mild smoking patients
Possibility to come to the dental office for dental treatments and follow-ups, as many times as necessary
Patient’s acceptance to participate in the study, with signed informed consent

**Table 2:** Exclusion criteria for patients in the study.

Non-vital, fractured, restored teeth with abfraction lesions
Absent teeth or incorrect restorations on the antagonistic teeth to the selected tooth with abfraction lesion
Rampant caries
Poor oral hygiene
Acute or chronic periodontal diseases
Endodontic conditions
Patients diagnosed with or in treatment for dento-maxillary anomalies
Occlusal wear, abrasion, erosion, parafunctions Allergic responses to any of the dental materials
Heavy smoker patients
Acute and chronic systemic disorders/diseases
Mental disability
Missing data
Uncooperative patients with lack of compliance
Pregnant and lactating women
Patients who refused to be included in the study

**Table 3:** Samples of patients (*n* = 53).

	1st Group (Only Fillings of Abfraction Lesions)	2nd Group (Fillings of Abfraction Lesions and Mouth Guard)
No. of patients	28	25
Age (mean ± years)	48.5 ± 10.5	48.5 ± 10.5
Gender M/F	12/16	14/11
No. of abfraction lesions	114	105
Localization of abfraction lesions	61 maxillary: 19 canines, 42 premolars 53 mandibular premolars	57 maxillary: 15 canines, 42 premolars 48 mandibular premolars

**Table 4:** Modified USPHS criteria for direct clinical evaluation of restorations [32,33].

Criteria	Rating	Aspect	Method
Color match	Alpha (A)	The restoration arises to fit in with the shade and translucency of adjacent hard tooth structures	Visual inspection
	Bravo (B)	The restoration does not fit in with the shade and translucency of adjacent tooth hard structures, but the discrepancy is within the standard scale of the tooth shades	Visual inspection
	Charlie (C)	The restoration does not fit in with the shade and translucency of neighboring teeth structures; discrepancy is outside of the standard scale of the tooth shades and translucency	Visual inspection
Marginal discoloration	Alpha (A)	No visible marginal discoloration between the restorative material color and of the neighboring hard tooth structure	Visual inspection
	Bravo (B)	Marginal discoloration present between the hard tooth tissues and restorative material, without penetration in pulpal direction; possible to be polished	Visual inspection
	Charlie (C)	Marginal modification of color shade between the tooth structure and restoration; infiltration in pulpal direction	Visual inspection
Surface texture	Alpha (A)	Restoration surface without any defects	Explorer
	Bravo (B)	Restoration surface with gritty texture	Explorer
	Charlie (C)	Coarse surface of restoration	Explorer
Anatomic contour/ form	Alpha (A)	The restoration is in the normal anatomic form, or is only a little flattened/over-contoured; by the examination of passing between the tooth tissues and the restoration margins, the tip of the dental explorer, positioned tangentially across the restoration material, does not experience two angles at the same time	Visual inspection and explorer
	Bravo (B)	The restoration surface present an evident concavity; in the examination of passing between the tooth tissues and the restoration margins, the tip of the dental explorer, positioned tangentially across the restoration material, does not experience two angles at the same time, but the dentin or base of filling is not exposed	Visual inspection and explorer
	Charlie (C)	There is present a loss of restorative material, and the surface of filling present an evident concavity; the base material of filling and/or the dentin are exposed	Visual inspection and explorer
Marginal adaptation /integrity	Alpha (A)	No noticeable evidence of a crack along the limit between the filling material and tooth structure	Explorer
	Bravo (B)	Noticeable evidence of a crack along the limit between the filling material and tooth structure, in which the dental explorer penetrates or catches	Explorer
	Charlie (C)	The explorer penetrates in the cracked area, which extends to the dento-enamel junction, and dentin or base filling material is exposed	Explorer
Secondary/recurrent caries	Alpha (A)	The restoration is in continuation of the existing anatomic tooth form	Visual inspection and explorer
	Charlie (C)	There are visual evidences of darkish discoloration in adjoining area of the restoration; caries lesions are present and connected with the filling material	Visual inspection and explorer
Retention of restoration/fracture	Alpha (A)	Restoration is intact and fully retained/fully preserved	Visual inspection
	Bravo (B)	Restoration is partially retained, but some broken part of the filling material is present	Visual inspection
	Charlie (C)	Restorative material was lost in totality	Visual inspection

**Table 5:** Results of the assessed criteria in the 1st group of patients (*n* = 28, with 114 esthetic fillings of abfraction lesions).

Criteria		Baseline			At 2 Months			At 6 Months			At 12 Months			At 18 Months			At 24 Months		
		FuB 38 Fillings	OmF 37 Fillings	BeaII 39 Fillings	FuB 38 Fillings	OmF 37 Fillings	BeaII 39 Fillings	FuB 38 Fillings	OmF 37 Fillings	BeaII 39 Fillings	FuB 38 Fillings	OmF 37 Fillings	BeaII 39 Fillings	FuB 38 Fillings	OmF 37 Fillings	BeaII 39 Fillings	FuB 38 Fillings	OmF 37 Fillings	BeaII 39 Fillings
*Color match/* *stability*	**A**: *n* (%)	*38* 100%	*37* 100%	*39* 100%	*38* 100%	*37* 100%	*39* 100%	*37* 100%	*37* 100%	*37* 100%	*37* 97.36%	*37* 100%	*37* 94.87%	*36* 94.73%	*37* 100%	*37* 94.87%	*34* 89.47%	*34* 91.89%	*36* 92.30%
	**B**: *n* (%)	*-*	*-*	*-*	*-*	*-*	*-*	*-*	-	*-*	*1* 2.63%	*-*	*2* 5.12%	*2* 5.26%	*-*	*2* 5.12%	*3* 7.89%	*3* 8.10%	*2* 5.12%
	**C**: *n* (%)	*-*	*-*	*-*	*-*	*-*	*-*	*-*	*-*	*-*	*-*	*-*	*-*	-	*-*	*-*	*1* 2.63%	*-*	*1* 2.56%
*Marginal discoloration*	**A**: *n* (%)	*38* 100%	*37* 100%	*39* 100%	*38* 100%	*37* 100%	*39* 100%	*38* 100%	*37* 100%	*39* 100%	*37* 97.36%	*37* 100%	*38* 97.43%	*37* 97.36%	*37* 100%	*37* 94.87%	*35* 92.10%	*35* 94.59%	*37* 94.87%
	**B**: *n* (%)	*-*	*-*	*-*	*-*	*-*	*-*	*-*	*-*	*-*	*1* 2.63%	*-*	*1* 2.56%	*1* 2.63%	*-*	*2* 5.12%	*3* 7.89%	*2* 5.40	*2* 5.12%
	**C**: *n* (%)	*-*	*-*	*-*	*-*	*-*	*-*	*-*	*-*	*-*	*-*	*-*	*-*	*-*	*-*	-	*-*	-	-
*Surface texture*	**A**: *n* (%)	*38* 100%	*37* 100%	*39* 100%	*38* 100%	*37* 100%	*39* 100%	*38* 100%	*37* 100%	*39* 100%	*37* 97.36%	*37* 100%	*38* 97.43%	*36* 94.73%	*37* 0%	*38* 97.43%	*34* 89.47%	*35* 94.59%	*36* 92.30%
	**B**: *n* (%)	*-*	*-*	*-*	*-*	*-*	*-*	*-*	*-*	*-*	*1* 2.63%	*-*	*1* 2.56%	*2* 5.26%	-	*1* 2.56%	*3* 7.89%	*2* 5.40%	*3* 7.69%
	**C**: *n* (%)	*-*	*-*	*-*	*-*	*-*	*-*	*-*	*-*	*-*	*-*	*-*	*-*	-	-	*-*	*1* 2.63%	-	-
*Anatomical* *contour* /*form*	**A**: *n* (%)	*38* 100%	*37* 100%	*39* 100%	*38* 100%	*37* 100%	*39* 100%	*38* 100%	*37* 100%	*39* 100%	*37* 97.36%	*36* 97.29%	*38* 97.43%	*36* 92.1 -	*36* 97.29%	*38* 97.43%	*35* 92.10%	*36* 97.29%	*37* 94.87%
	**B**: *n* (%)	*-*	*-*	*-*	*-*	*-*	*-*	*-*	*-*	*-*	*1* 2.63%	*1* 2.7%	*1* 2.56%	*2* 5.26%	*1* 2.7%	*1* 2.56%	*2* 5.26%	*1* 2.7%	*2* 5.12%
	**C**: *n* (%)	*-*	*-*	*-*	*-*	*-*	*-*	*-*	*-*	*-*	-	-	-	*-*	-	-	*1* 2.63%	-	*-*
*Marginal* *adaptation/* *integrity*	**A**: *n* (%)	*38* 100%	*37* 100%	*39* 100%	*38* 100%	*37* 100%	*39* 100%	*38* 100%	*37* 100%	*39* 100%	*37* 97.36%	*37* 0%	*38* 97.43%	*35* 92.1 -	*36* 97.29%	*37* 94.87%	*34* 89.47%	*35* 94.59%	*36* 92.3%
	**B**: *n* (%)	*-*	*-*	*-*	*-*	*-*	*-*	*-*	*-*	*-*	*1* 2.63%	*-*	*1* 2.56%	*1* 2.63%	*1* 2.7%	*1* 2.56%	*3* 7.89%	*2* 5.40%	*2* 5.12%
	**C**: *n* (%)	*-*	*-*	*-*	*-*	*-*	*-*	*-*	*-*	*-*	*-*	*-*	*-*	*1* 2.63%	-	*1* 2.56%	*1* 2.63%	-	*1* 2.56%
*Secondary*/ *recurrent caries*	**A**: *n* (%)	*38* 100%	*37* 100%	*39* 100%	*38* 100%	*37* 100%	*39* 100%	*38* 100%	*37* 100%	*39* 100%	*37* 97.36%	*37* 0%	*38* 97.43%	*36* 94.73%	*36* 97.29%	*38* 97.43%	*36* 94.73%	*36* 97.29%	*37* 94.87%
	**C**: *n* (%)	*-*	*-*	*-*	*-*	*-*	*-*	*-*	*-*	*-*	*1* 2.63%	-	*1* 2.56%	*2* 5.26%	*1* 2.7%	*1* 2.56%	*2* 5.26%	*1* 2.7%	*2* 5.12%
*Retention of restoration/* *fracture*	**A**: *n* (%)	*38* 100%	*37* 100%	*39* 100%	*38* 100%	*37* 100%	*39* 100%	*38* 100%	*37* 100%	*39* 100%	*36* 94.73%	*37* 0%	*38* 97.43%	*35* 92.1 -	*36* 97.29%	*38* 97.43%	*34* 89.47%	*35* 94.59%	*36* 92.3%
	**B**: *n* (%)	*-*	*-*	*-*	*-*	*-*	*-*	*-*	*-*	*-*	*1* 2.63%	-	*1* 2.56%	*1* 2.63%	*1* 2.7%	*1* 2.56%	*2* 5.26%	*2* 5.40%	*2* 5.12%
	**C**: *n* (%)	*-*	*-*	*-*	*-*	*-*	*-*	*-*	*-*	*-*	*-*	-	-	*2* 5.26%	-	-	*2* 5.26%	-	*1* 2.56%

**FuB**—Fuji Bulk capsules; **OmF**—Omnichroma Flow; **BeaII**—Beautifil^®^ II; Alpha (**A**)—excellent results; Bravo (**B**)—acceptable results; Charlie (**C**)—unacceptable results and replacement of the restoration is necessary.

**Table 6:** Results of the assessed criteria in the 2nd group of patients (*n* = 25, with 105 esthetic fillings and thermoformed mouthguards).

Criteria		Baseline			At 2 Months			At 6 Months			At 12 Months			At 18 Months			At 24 Months		
		FuB 34 Fillings	OmF 36 Fillings	BeaII 35 Fillings	FuB 34 Fillings	OmF 36 Fillings	BeaII 35 Fillings	FuB 34 Fillings	OmF 36 Fillings	BeaII 35 Fillings	FuB 34 Fillings	OmF 36 Fillings	BeaII 35 Fillings	FuB 34 Fillings	OmF 36 Fillings	BeaII 35 Fillings	FuB 34 Fillings	OmF 36 Fillings	BeaII 35 Fillings
*Color match/* *stability*	**A**: *n* (%)	*34* 100%	*36* 100%	*35* 100%	*34* 100%	*36* 100%	*35* 0%	*34* 100%	*36* 100%	*35* 100%	*34* 100%	*36* 100%	*35* 100%	*32* 94.11%	*35* 97.22%	*33* 94.28%	*30* 88.23%	*34* 94.44%	*32* 91.42%
	**B**: *n* (%)	*-*	*-*	-	*-*	*-*	-	*-*	*-*	-	*-*	*-*	-	*2* 5.88%	*1* 2.77%	*2* 5.71%	*3* 8.82%	*2* 5.55%	*2* 5.71%
	**C**: *n* (%)	*-*	*-*	-	*-*	*-*	-	*-*	*-*	-	*-*	*-*	-	*-*	-	-	*1* 2.94%	-	*1* 2.85%
*Marginal discoloration*	**A**: *n* (%)	*34* 100%	*36* 100%	*35* 100%	*34* 100%	*36* 100%	*35* 100%	*34* 100%	*36* 100%	*35* 100%	*34* 100%	*36* 100%	*35* 100%	*32* 94.11%	*36* 100%	*33* 94.28%	*31* 91.17%	*34* 94.44%	*32* 91.42%
	**B**: *n* (%)	*-*	*-*	- -	*-*	*-*	- -	*-*	*-*	- -	*-*	*-*	*-*	*2* 5.88%	*-*	*2* 5.71%	*3* 8.82%	*2* 5.55%	*3* 8.57%
	**C**: *n* (%)	*-*	*-*	*-* -	*-*	*-*	*-* -	*-*	*-*	*-* -	*-*	*-*	*-*	*-*	-	-	*-*	-	*-*
*Surface texture*	**A**: *n* (%)	*34* 100%	*36* 100%	*35* 100%	*34* 100%	*36* 100%	*35* 100%	*34* 100%	*36* 100%	*35* 100%	*34* 100%	*36* 100%	*35* 100%	*32* 94.11%	*35* 97.22%	*33* 94.28%	*30* 88.23%	*34* 94.44%	*33* 94.28%
	**B**: *n* (%)	*-*	*-*	*-*	*-*	*-*	*-*	*-*	*-*	*-*	*-*	*-*	*-*	*2* 5.88%	*1* 2.77%	*2* 5.71%	*3* 8.82%	*2* 5.55%	*2* 5.71%
	**C**: *n* (%)	*-*	*-*	*-*	*-*	*-*	*-*	*-*	*-*	*-*	*-*	*-*	*-*	*-*	-	-	*1* 2.94%	-	-
*Anatomical* *contour/form*	**A**: *n* (%)	*34* 100%	*36* 100%	*35* 100%	*34* 100%	*36* 100%	*35* 100%	*34* 100%	*36* 100%	*35* 100%	*34* 100%	*36* 100%	*35* 100%	*33* 97.05%	*35* 97.22%	*33* 94.28%	*30* 88.23%	*34* 94.44%	*33* 94.28%
	**B**: *n* (%)	*-*	*-*	-	*-*	*-*	- -	*-*	*-*	-	*-*	*-*	*-*	*1* 2.94%	*1* 2.77%	*2* 5.71%	*3* 8.82%	*2* 5.55%	*2* 5.71%
	**C**: *n* (%)	*-*	*-*	-	-	-	-	*-*	*-*	*-*	*-*	*-*	*-*	-	*-*	*-*	*1* 2.94%	*-*	*-*
*Marginaladaptation*/ *integrity*	**A**: *n* (%)	*34* 100%	*36* 100%	*35* 100%	*34* 100%	*36* 100%	*35* 100%	*34* 100%	*36* 100%	*35* 100%	*34* 100%	*36* 100%	*35* 100%	*31* 91.17%	*35* 97.22%	*33* 94.28%	*31* 91.17%	*34* 94.44%	*32* 91.42%
	**B**: *n* (%)	*-*	*-*	*-*	*-*	*-*	*-*	*-*	*-*	*-*	*-*	*-*	*-*	*2* 5.88%	*1* 2.77%	*2* 5.71%	*2* 5.88%	*2* 5.55%	*3* 8.57%
	**C**: *n* (%)	*-*	*-*	*-*	*-*	*-*	*-*	*-*	*-*	*-*	*-*	*-*	*-*	*1* 2.94%	*-*	*-*	*1* 2.94%	*-*	*-*
*Secondary*/ *recurrent caries*	**A**: *n* (%)	*34* 100%	*36* 100%	*35* 100%	*34* 100%	*36* 100%	*35* 100%	*34* 100%	*36* 100%	*35* 100%	*34* 100%	*36* 100%	*35* 100%	*33* 97.06%	*36* 100%	*35* 100%	*32* 94.11%	*35* 97.22%	*33* 94.28%
	**C**: *n* (%)	*-*	*-*	*-*	*-*	*-*	*-*	*-*	*-*	*-*	*-*	*-*	*-*	*1* 2.94%	-	-	*2* 5.88%	*1* 2.77%	*2* 5.71%
*Retention of restoration*/ *fracture*	**A**: *n* (%)	*34* 100%	*36* 100%	*35* 100%	*34* 100%	*36* 100%	*35* 100%	*34* 100%	*36* 100%	*35* 100%	*34* 100%	*36* 100%	*35* 100%	*31* 91.17%	*35* 97.22%	*32* 91.42%	*31* 91.17%	*34* 94.44%	*32* 91.42%
	**B**: *n* (%)	*-*	*-*	*-*	*-*	*-*	*-*	*-*	*-*	*-*	*-*	*-*	*-*	*2* 5.88%	*1* 2.77%	*3* 8.57%	*1* 2.94%	*2* 5.55%	*2* 5.71%
	**C**: *n* (%)	*-*	*-*	*-*	*-*	*-*	*-*	*-*	*-*	*-*	*-*	*-*	*-*	*1* 2.94%	-	-	*2* 5.88%	-	*1* 2.85%

**FuB**—Fuji Bulk capsules; **OmF**—Omnichroma Flow; **BeaII**—Beautifil^®^ II; Alpha (**A**)—excellent results; Bravo (**B**)—acceptable results; Charlie (**C**)—unacceptable results and replacement of the restoration is necessary.

**Table 7:** Comparison of (B), (C), and (B + C) *p* values in both groups of patients, at all assessments.

Rating for Color Match/Stability	Sum of Undesirable Scores in all Assessments (*n*; %)				*p*
	*1st Group of Patients*		*2nd Group of Patients*		
***Color match/stability criteria***					
**B**: *n* (%)	**FuB** 38 fillings	*7*; (18.42%)	**FuB** 34 fillings	*5*; (13.15%)	*0.65*
**C**: *n* (%)		*1*; (2.63%)		*1*; (2.63%)	*-*
Cumulative undesirable scores		*8*; (21.05%)		*6*; (15.78%)	*0.65*
**B**: *n* (%)	**OmF** 37 fillings	*3*; (8.10%)	**OmF** 36 fillings	*2*; (5.55%)	*0.223*
**C**: *n* (%)		*0*; (0%)		*0*; (0%)	*-*
Cumulative undesirable scores		*3*; (8.10%)		*2*; (5.55%)	*0.223*
**B**: *n* (%)	**BeaII** 39 fillings	*6*; (15.38%)	**BeaII** 35 fillings	*4*; (10.25%)	*0.709*
**C**: *n* (%)		*1*; (2.56%)		*1*; (2.85%)	*-*
Cumulative undesirable scores		*7*; (17.94%)		*5*; (12.82%)	*0.709*
***Marginal discoloration***					
**B**: *n* (%)	**FuB** 38 fillings	*5*; (13.15%)	**FuB** 34 fillings	*5*; (14.70%)	*0.233*
**C**: *n* (%)		*0*; (0%)		*0*; (0%)	*-*
Cumulative undesirable scores		*5*; (13.15%)		*5*; (14.70%)	*0.172*
**B**: *n* (%)	**OmF** 37 fillings	*2*; (5.40%)	**OmF** 36 fillings	*2*; (5.55%)	*0.386*
**C**: *n* (%)		*0*; (0%)		*0*; (0%)	*-*
Cumulative undesirable scores		*2*; (5.40%)		*2*; (5.55%)	*0.385*
**B**: *n* (%)	**BeaII** 39 fillings	*5*; (12.82%)	**BeaII** 35 fillings	*5*; (14.28%)	*0.361*
**C**: *n* (%)		*0*; (%)		*0*; (0%)	*-*
Cumulative undesirable scores		*5*; (12.82%)		*5*; (14.28%)	*0.361*
***Surface texture***					
**B**: *n* (%)	**FuB** 38 fillings	*6*; (15.78%)	**FuB** 34 fillings	*5*; (14.70%)	*0.659*
**C**: *n* (%)		*0*; (0%)		*1*; (2.94%)	*-*
Cumulative undesirable scores		*6*; (15.78%)		*6*; (17.64%)	*0.659*
**B**: *n* (%)	**OmF** 37 fillings	*2*; (5.40%)	**OmF** 36 fillings	*3*; (8.33%)	*0.386*
**C**: *n* (%)		*0*; (0%)		*0*; (0%)	*-*
Cumulative undesirable scores		*2*; (5.40%)		*3*; (8.33%)	*0.386*
**B**: *n* (%)	**BeaII** 39 fillings	*5*; (12.82%)	**BeaII** 35 fillings	*4*; (11.42%)	*0.082*
**C**: *n* (%)		*0*; (%)		*0*; (0%)	*-*
Cumulative undesirable scores		*5*; (12.82%)		*4*; (11.42%)	*0.082*
***Anatomical*** ***contour/*** ***form***					
**B**: *n* (%)	**FuB** 38 fillings	*5*; (13.15%)	**FuB** 34 fillings	*4*; (11.76%)	*0.233*
**C**: *n* (%)		*1*; (2.63%)		*1*; (2.940%)	*-*
Cumulative undesirable scores		*6*; (15.78%)		*5*; (14.70%)	*0.405*
**B**: *n* (%)	**OmF** 37 fillings	*3*; (8.1%)	**OmF** 36 fillings	*3*; (8.33%)	*0.171*
**C**: *n* (%)		*0*; (0%)		*0*; (0%)	*-*
Cumulative undesirable scores		*3*; (8.1%)		*3*; (8.33%)	*0.171*
**B**: *n* (%)	**BeaII** 39 fillings	*4*; (10.25%)	**BeaII** 35 fillings	*4*; (11.42%)	*0.136*
**C**: *n* (%)		*0*; (%)		*0*; (0%)	*-*
Cumulative undesirable scores		*4*; (10.25%)		*4*; (11.42%)	*0.136*
***Marginal*** ***adaptation/*** ***integrity***					
**B**: *n* (%)	**FuB** 38 fillings	*5*; (13.15%)	**FuB** 34 fillings	*4*; (11.76%)	*0.082*
**C**: *n* (%)		*2*; (5.26%)		*2*; (5.88%)	*1*
Cumulative undesirable scores		*7*; (19.44%)		*6*; (17.64%)	*0.172*
**B**: *n* (%)	**OmF** 37 fillings	*3*; (8.1%)	**OmF** 36 fillings	*3*; (8.33%)	*1*
**C**: *n* (%)		*0*; (0%)		*0*; (0%)	*-*
Cumulative undesirable scores		*3*; (8.1%)		*3*; (8.33%)	*1*
**B**: *n* (%)	**BeaII** 39 fillings	*5*; (12.82%)	**BeaII** 35 fillings	*5*; (14.28%)	*0.233*
**C**: *n* (%)		*2*; (5.88%)		*0*; (0%)	*-*
Cumulative undesirable scores		*7*; (17.94%)		*5*; (14.28%)	*0.659*
***Secondary/recurrent caries***					
**C**: *n* (%)	**FuB** 38 fillings	*5*; (13.15%)	**FuB** 34 fillings	*3*; (8.82%)	*0.709*
Cumulative undesirable scores		*5*; (13.15%)		*3*; (8.82%)	*0.709*
**C**: *n* (%)	**OmF** 37 fillings	*2*; (5.40%)	**OmF** 36 fillings	*1*; (2.77%)	*0.34*
Cumulative undesirable scores		*2*; (5.40%)		*1*; (2.77%)	*0.34*
**C**: *n* (%)	**BeaII** 39 fillings	*4*; (10.25%)	**BeaII** 35 fillings	*2*; (5.71%)	*0.248*
Cumulative undesirable scores		*4*; (10.25%)		*2*; (5.71%)	*0.248*
***Retention of restoration/fracture***					
**B**: *n* (%)	**FuB** 38 fillings	*4*; (10.52%)	**FuB** 34 fillings	*3*; (8.82%)	*0.709*
**C**: *n* (%)		*4*; (10.52%)		*3*; (8.82%)	*0.248*
Cumulative undesirable scores		*8*; (21.05%)		*6*; (17.64%)	*0.329*
**B**: *n* (%)	**OmF** 37 fillings	*3*; (8.1%)	**OmF** 36 fillings	*3*; (8.33%)	*1*
**C**: *n* (%)		*0*; (0%)		*0*; (0%)	*-*
Cumulative undesirable scores		*3*; (8.1%)		*3*; (8.33%)	*0.368*
**B**: *n* (%)	**BeaII** 39 fillings	*4*; (10.25%)	**BeaII** 35 fillings	*5*; (14.28%)	*0.233*
**C**: *n* (%)		*1*; (2.56%)		*1*; (2.85%)	*-*
Cumulative undesirable scores		*5*; (12.82%)		*6*; (17.14%)	*0.136*

**FuB**—Fuji Bulk capsules; **OmF**—Omnichroma Flow; **BeaII**—Beautifil^®^ II; Alpha (**A**)—excellent results; Bravo (**B**)—acceptable results; Charlie (**C**)—unacceptable results and replacement of the restoration is necessary; **B** and **C** ratings are considered undesirable results.

## Data Availability

Not applicable.
